# Sequence Analysis and Identification of New Isoform of EP4 Receptors in Different Atlantic Salmon Tissues (*Salmo salar* L.) and Its Role in PGE2 Induced Immunomodulation *In Vitro*

**DOI:** 10.1371/journal.pone.0120483

**Published:** 2015-04-02

**Authors:** Tz Chun Guo, Amr Ahmed Abdelrahim Gamil, Melanie Koenig, Øystein Evensen

**Affiliations:** 1 Norwegian University of Life Sciences, Faculty of Veterinary Medicine and Biosciences, Sea Lice Research Centre, P.O. Box 8146 Dep., 0033 Oslo, Norway; 2 European Neuroscience Institute, Synaptic Vesicle Dynamics, 37077 Göttingen, Germany; Medical School of Hannover, GERMANY

## Abstract

PGE2 plays an important role in a broad spectrum of physiological and pathological processes mediated through a membrane-bound G protein-coupled receptor (GPCR) called EP receptor. In mammals, four subtypes of EP receptor (EP 1-4) are identified and each of them functions through different signal transduction pathways. Orthologous EP receptors have also been identified in other non-mammalian species, such as chicken and zebrafish. EP4 is the only identified PGE2 receptor to date in Atlantic salmon but its tissue distribution and function have not been studied in any detail. In this study, we first sequenced EP4 receptor in different tissues and found that the presence of the 3nt deletion in the 5’ untranslated region was accompanied by silent mutation at nt 668. While attempting to amplify the same sequence in TO cells (an Atlantic salmon macrophage-like cell line), we failed to obtain the full-length product. Further investigation revealed different isoform of EP4 receptor in TO cells and we subsequently documented its presence in different Atlantic salmon tissues. These two isoforms of EP4 receptor share high homology in their first half of sequence but differ in the second half part with several deletion segments though the final length of coding sequence is the same for two isoforms. We further studied the immunomodulation effect of PGE2 in TO cells and found that PGE2 inhibited the induction of CXCL-10, CCL-4, IL-8 and IL-1β genes expression in a time dependent manner and without cAMP upregulation.

## Introduction

Prostaglandins (PG) are arachidonic acid (AA) metabolites that serve several biological and physiological functions. They are produced when AA is released from the plasma membrane by phospholipases and metabolized by cyclooxygenases (also known as PG synthases) [1]. PGs are ubiquitously produced in the body. They act in an autocrine or paracrine manner by activating intracellular signaling cascade through rhodopsin-like 7 transmembrane-spanning G protein coupled receptors [2]. Four different bioactive PGs are found; PGE_2_, prostacycline (PGI_2_), PGD_2_ and PGF_2α_. Among these different PGs, PGE_2_ has the broadest range of biological actions and is known for its diverse role in inflammation, immune response and reproduction [3–6].

As for other PGs, PGE_2_ exerts its function by binding to its specific receptors leading to activation of intracellular signaling and gene transcription. Four different PGE_2_ receptors have been identified designated as EP1, EP2, EP3 and EP4 receptors. While these receptors are highly conserved between mammalian species, significant differences in the structure and pharmacology exist between the different subtypes [7]. EP4 receptor is characterized by its long carboxyl terminus which is important for agonist-induced desensitization [8]. EP3 receptors are distinguished by the presence of multiple variants generated by alternative splicing of the C terminus [9].

Each of the EP receptors has distinct signal transduction properties and tissue and cellular distribution. In mice, for example, EP3 and EP4 are the most widely distributed subtypes whereas EP2 is the least abundant [10]. The signaling cascade induced by EP1 is coupled to phospholipase C/l-1,4,5-trisphosphate signaling pathway, and leads to mobilization of intracellular calcium [11], while signaling through EP2 and EP4 receptor results in increased intracellular cyclic AMP (cAMP) levels via coupling to G_s_ proteins [12]. Signaling through EP3, on the other hand, is coupled to a G_i_ protein and results in reduction in intracellular cAMP levels [13]. However, activation of at least one EP3 splice variant can also lead to increased cAMP levels, suggesting functional coupling to a G_s_ protein [14]. This diversity in receptor and signal transduction property is largely responsible for the pleiotropic effect of PGE_2_. For example, EP2 and EP4 can mediate both pro- and anti-inflammatory effects while the responses induced by EP1 and EP3 are mostly pro-inflammatory [15].

Presence of PGE2 in the aquatic invertebrates and vertebrates has been reported since the 1970s [16,17]. Since then, investigators have studied different aspects of PGE response in several fish species. In contrast, detailed information about receptor subtypes and their functions are still lacking. The best-studied fish species with regard to EP4 receptors is zebrafish. Although all the four subtypes are found [18–20], basic information such as the tissue distribution and the functions they play is still lacking. Presence of multiple isoforms for EP2 and EP4 suggests that functional differences may exist between these receptors and their mammalian counterparts. Whether presence of multiple isoform for EP2 and EP4 is a common feature across fish species or rather specific to the zebrafish is not known. For Atlantic salmon, the only identified PGE2 receptor so far is EP4 [21]. In the present study we aimed to investigate the presence and distribution of EP4 receptor in Atlantic salmon and evaluate its immunomodulation effect *in vitro* using TO cells, a salmon macrophage-like cell line as a model. To our surprise, a different EP4 isoform was identified in TO cells and this sequence was shown to be present also in Atlantic salmon tissues. We further studied the immunomodulation inferred by PGE2 and showed the effect likely through a cAMP-independent pathway.

## Materials and Methods

### Cell culture

Chinook salmon embryonic cells (CHSE-214) [22], TO cells, a macrophage-like cell line originating from Atlantic salmon head kidney leukocytes [23], and Rainbow trout gonad cells (RTG-2) [24] were maintained at 20°C in L-15 glutamax media (Invitrogen) supplemented with 10% FBS (Invitrogen). During PGE2 stimulation experiment 50μg/mL gentamycin was additionally added to the media.

### RNA isolation and cDNA synthesis

Total RNA was isolated from TO cells, headkidney macrophages and different Atlantic salmon tissues. For TO cells and macrophages, RNA was extracted using RNeasy Plus mini kit (Qiagen) following the manufacturer’s protocol while a combination of Trizol and RNeasy mini kit procedures was used for the tissues. For the latter, approximately 30mg tissues were added to eppendorf tubes containing 1ml ISol-RNA Lysis Reagent (5Prime). Tissues were then homogenized in Mixer Mill MM301 homogenizer (Retsch) using stainless beads at 20HZ and homogenates were subjected to phase separation by adding 0.2ml chloroform. The aqueous phase was passed through gDNA column followed by RNA isolation using RNeasy Plus mini kit (Qiagen) following the manufacturer’s protocol. The obtained RNA concentration was determined by spectrophotometry using Nanodrop ND1000 (Thermo Scientific).

Following RNA isolation, 1μg RNA was used for cDNA synthesis using Transcription First Strand cDNA Synthesis Kit (Roche) using both oligo (dT) and random hexamer primers, following the manufacturer’s protocol. The experiments/procedures reported herein have been conducted in accordance with the laws and regulations controlling experiments/procedures in live animals in Norway, i.e. the Animal Welfare Act of December 20th 1974, No 73, chapter VI sections 20–22 and the Regulation on Animal Experimentation of January 15th 1996. In addition, Norway has signed and ratified The European Convention for the protection of Vertebrate Animals used for Experimental and other Scientific Purposes of March 18th 1986. The Norwegian legislation conforms in all respects with the basic requirements of this Convention and guidelines prepared in pursuance of it. The National Animal Research Authority (NARA) authority and the Norwegian University of Life Sciences´ IACUC committee have approved the entire study.

### Amplification, cloning and sequencing of the full-length EP4 receptors in different tissues

To amplify the full-length (FL) EP4 from Atlantic salmon tissue, cDNA was diluted 1:5, 4μl of each was used for PCR amplification (40 cycles) using Accustart Taq DNA Polymerase HiFi (Quanta Bioscience, Gaitherburg, MD, USA) and 5pmol FL-EP4a forward and reverse primers (Table 1). The cycling conditions were as follow: 94°C for 30sec, 60°C for 1min, 68°C for 2,5min.

**Table 1:** List of primers used for PCR and cloning.

Name	Sequence	Used for	Accession no
O-EP4-F1-For TO-EP4-F1-Rev	5´-ACAAAAACACTTCGGATAGTCAAAAACC-3´ 5´-GTATCTCTCTATGGACATGGCACAG-3´	PCR	-
TO-EP4-F2-For TO-EP4-F2-Rev	5´-CACCATCGCCACCTATGTGC-3´ 5´-GCAGATGAGAACAACCACGG-3´	PCR	-
TO-EP4-F3-For TO-EP4-F3-Rev	5´-CAACGATTGGCCGGTGCAG-3´ 5´-CTGTGACTTTTCTGTGTTATCC-3´	PCR	-
FL-EP4-For FL-EP4-Rev	5´-ACAAAAACACTTCGGATAGTCAAAAACC-3´ 5´-GGGACAAAGTTCACATTGTAGCC-3´	PCR	NM_001173955.1
As-EP4a-For As-EP4a-Rev	5´- GTCAACCCCATCCTTGACCC-3´ 5´- CAGCCCCCCTTTAGTCCCCT-3´	PCR	-
As-EP4b-For As-EP4b-Rev	5´-CACTGCGGAGGTGAAGGTC-3´ 5´-GGGGCCTAAAGTGCCCTTTTTG-3´	PCR	-
M13-For M13-Rev	5´-GTAAAACGACGGCCAG-3´ 5´-CAGGAAACAGCTATGAC-3´	Cloning/ sequencing	-
CXCL10-For CXCL10-Rev	5´-CAGGTGGGTCATTCTAAAGC-3´ 5´-CTTGGCAAATGGAGCTTCTG-3´	Realtime PCR	AJ417078.1
CCL4-For CCL4-Rev	5´-TGATCGTCAGATACCCAGAG-3´ 5´-GTTGATGTAGTCCTTCACCC-3´	Realtime PCR	NM_001123618.1
IL8-For IL8-Rev	5´-GCAGACGAATTGGTAGACTC-3´ 5´-TCTTCTTAATGACCCTCTTGAC-3´	Realtime PCR	NM_001140710.2
IL6-For IL6R-ev	5´-GGAGGAGTTTCAGAAGCCCG-3´ 5´-TGGTGGTGGAGCAAAGAGTCT-3´	Realtime PCR	NM_001124657

PCR products were cloned into pCR2.1 vector following the protocol provided with the TOPO TA cloning Kit (Invitrogen). The cloning reaction was transformed into *E*. *coli* Top 10 chemically competent cells (Invitrogen) and plated on LB agar medium containing 100 μg/ml Ampicillin. The positive clones were verified by PCR, using VWR Taq DNA Polymerase (VWR) and M13 primers (Table 1), and 10 colonies from each tissue were subsequently sent to GATC biotech for sequencing.

### Identification of a new isoform of EP4 gene in TO cells

Total RNAs were isolated from TO cells and cDNAs were synthesized as described above. Partial sequences of EP4 from TO cells were obtained by PCR using randomly designed primers (data not shown) based on the published Atlantic salmon EP4 sequence (NM_001173955.1). Finally, the nearly full length sequence except 5’ and 3’ end was obtained by amplifying three fragments using TO-EP4 primers (Table 1) and the PCR products were subsequently purified and sequenced by GATC biotech (Germany). To obtain the sequences for the 5’ and 3’ ends, 5’RACE-GSP (GTATCTCTCTAT GGACATGGCACAG) and 3’RACE-GSP (ATATACCAGCACACACATCAGTAATGA AC) primers were designed based on the sequence information obtained from PCR products and RACE PCR was performed using SMARTer RACE cDNA amplification kit (Clontech) according to the manufacturer’s instructions.

### Molecular phylogenetic analysis

The sequences of EP4 receptors from a number of species were obtained from GenBank: Humans (NM_000958), rat (NM_ 032076), zebrafish EP4a (NM_001039629.1), zebrafish EP4b (NM_001128367), zebrafish EP4c (NM_001281996), mouse (NM_001136079), dog (NM_00100 3054), chicken (NM_001081503), and cattle (NM_174589). The newly identified EP receptor for Atlantic salmon was included in the analysis. A phylogenetic tree was constructed with amino acid sequences of all species above using PhyML program (http://www.phylogeny.fr/).

### Isolation of headkidney macrophages

Macrophages were isolated from the headkidney using Percoll gradient as previously described [25]. Briefly, the tissue was passed through 100μm nylon mesh and collected in isolation medium (L-15-glutamax medium containing heparin and 2% FBS). The cell suspension was then layered on top of 37/54% Percoll gradient and centrifuged at 600g for 40 min at 4°C. The band of cells between the two Percoll layers were collected and washed once in L-15-glutamax medium containing 0.1 FBS and 100U/ml penicillin/streptomycin (Gibco). About 8x10^5^ cells/well were seeded in 6 wells plate and incubated overnight at 15°C in L15-glutamax medium containing 100U/ml penicillin/streptomycin and 20% FBS before being subjected to RNA extraction and cDNA synthesis as described above.

### Effect of PGE2 on LPS induced responses

TO cells were first starved in L-15 media (Invitrogen) containing 0.5%FBS for 12–15 hours and then incubated for one hour with different concentrations of PGE2 (Sigma Aldrich) prepared in L-15 media supplemented with 10% FBS and 50 μg/ml gentamycin. When EP4 antagonist L161,982 (Santa Cruz Biotech) was used 5μM was added to the cells 45min before the incubation with PGE2. Cells were subsequently stimulated with 50μg LPS (sigma) and harvested at different times post stimulation. Total RNA was then isolated followed by cDNA synthesis as already described above except that 400ng total RNA was used for cDNA synthesis. Changes in gene expression were assessed by real time PCR performed in 96 well plates using LightCycler 480 (Roche). For each reaction, 4μl cDNA was mixed with 10pmol gene specific primers and 10μl LightCycler 480 SYBR green I master mix (Roche). The final concentration was adjusted to 20μl using RNase free water. The genes assessed and the sequences of primers used in the reactions are provided in Table 1. The cycling conditions were as follows: denaturation 94°C for 10 sec; Annealing 60°C for 20 sec; elongation 72°C for 10 sec. The results were analyzed by the ΔΔCT relative quantification approach [26] using β-actin as reference gene.

### cAMP assay

TO, RTG-2 and CHSE-214 cells were seeded in 6 wells plate (Corning) and incubated at 20°C until confluency. At the time of experiment, cells were treated with 10μM PGE2 (Sigma Aldrich), 100μM forskolin (Sigma Aldrich), or left untreated. Cells were then incubated at 20°C for 1 hour. Prior to the assay, cells were washed three times in cold PBS and subsequently lysed in Cell Lysis Buffer provided in the kit. The amount of cAMP was measured using the cAMP parameter assay kit (R&D systems). The procedure was performed following the manufacturer’s instructions. As this assay is based on competitive binding technique, the readout value of OD450 is inversely proportional to the concentration of cAMP.

#### Software

Nucleotides and amino acid sequences of EP4 were aligned by CLC Workbench. Phylogenetic tree was also created by the same software. One-way ANOVA followed by Tukey´s multiple comparisons test was performed using GraphPad Prism version 6.00, GraphPad Software, La Jolla California USA.

## Results

### Sequence analysis in different tissues

Sequence analysis revealed very high similarity between the published EP4 sequence (GenBank: NM_001173955.1) and the sequences obtained from different tissues. However, a 3 nucleotide (nt) deletion was found in the 5’ untranslated region (UTR) at nt 228–230 in all tissues except the skin. Presence of the 3nt deletion was accompanied by silent mutation at nt 668. Additionally, some of the clones from the muscle had mutation at nt 448 from thymine to cytosine resulting in a Leu61Pro mutation. This mutation was only detected when the 5’UTR deletion was abscent.

### Two isoforms of EP4 are found in Atlantic salmon

**Fig 1:**
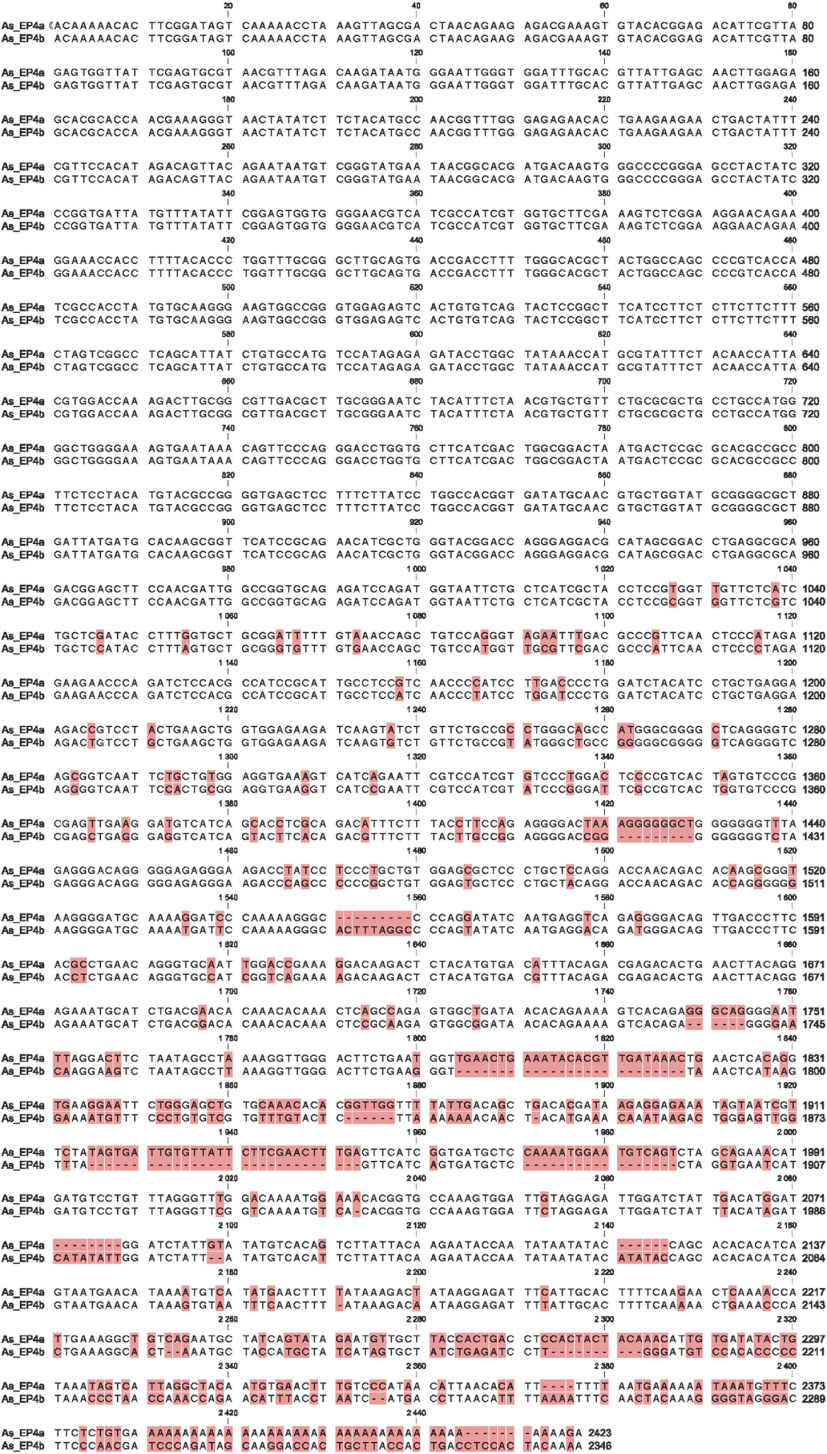
Nucleotide sequences of two EP4 subtypes. Comparison of nucleotide sequence of two subtypes of EP4 gene identified in Atlantic salmon.

**Fig 2:**
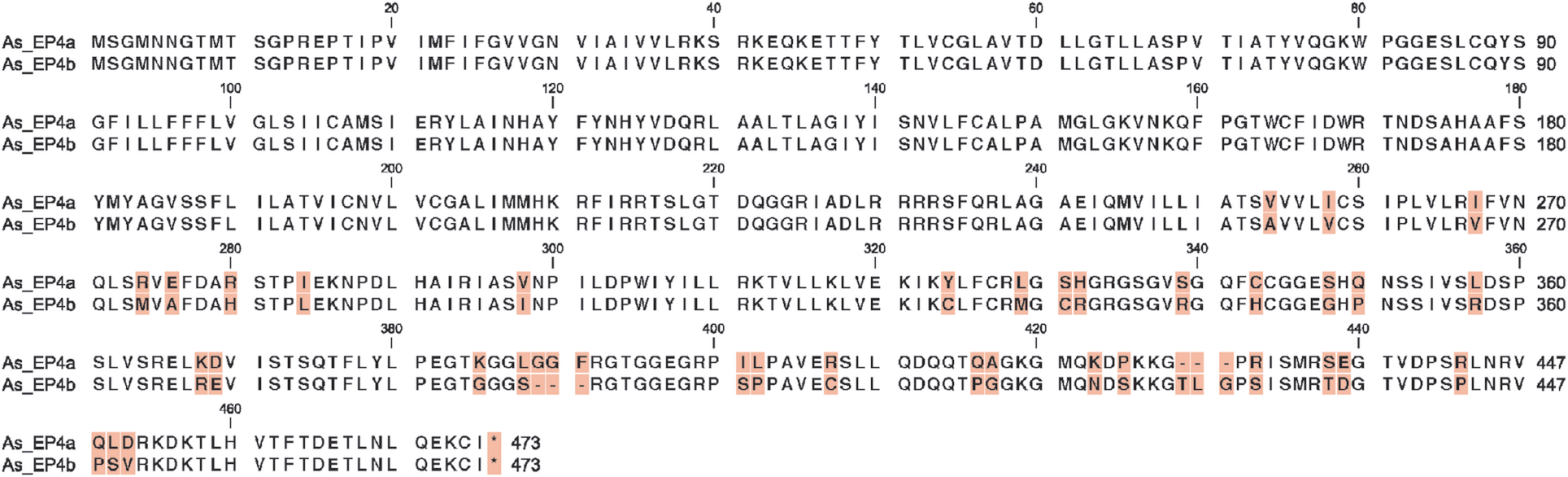
Amino acid sequence of two EP4 variants in salmon. Comparison of amino acid sequence of two subtypes of EP4 gene identified in Atlantic salmon.

**Fig 3:**
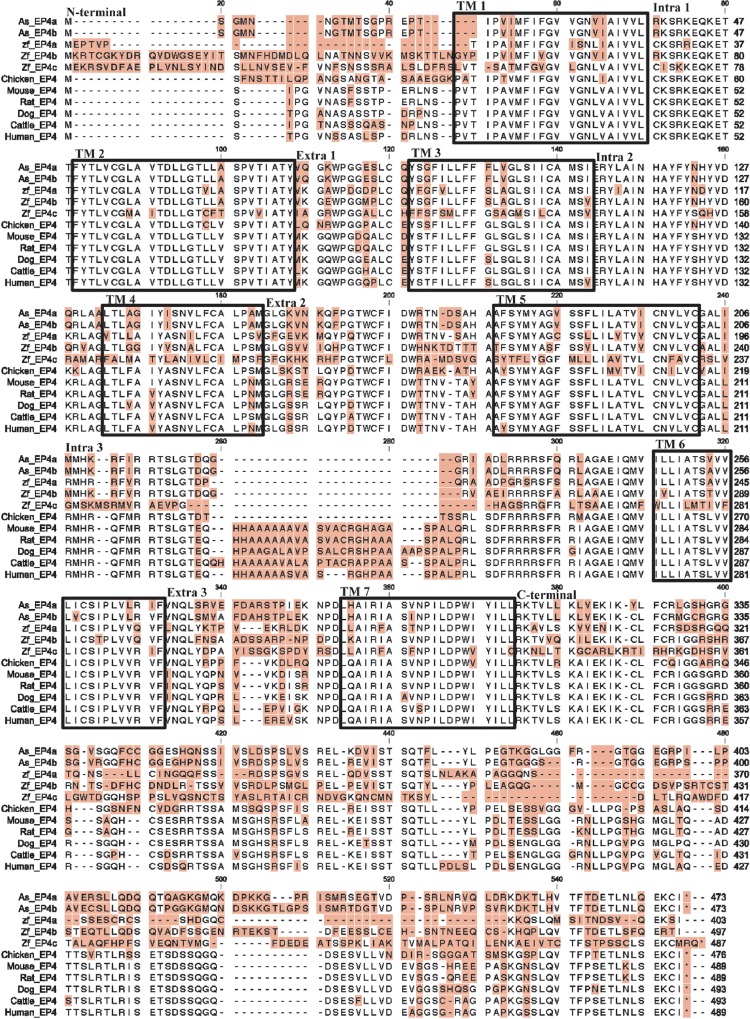
Alignment of EP4 amino acid sequence in different species. Differences in amino acids are highlighted by shading. Putative transmembrane domains (TM) 1–7 are shown by the rectangular boxes. Intracellular domains (Intra) 1–3, extracellular (Extra) domains 1–3, N-and C-terminal domains are noted in the beginning of the sequences.

While the full length EP4 sequence was easily obtained from several tissues, it could not be amplified from TO cells. We obtained the first half of EP4 when reverse primers were designed in the middle of the published EP4 sequence (data not shown). For the second part of the sequence, we designed several primers using published Atlantic salmon EP4 sequence and based on the fragmented sequences obtained, three PCR fragments constituting most of the sequence of EP4 gene in TO cells were amplified (excluding the 5’ and 3’ end sequences). The sequence for the 5’ and 3’ ends were then attained by 5’ and 3’ RACE as described in materials and methods. The result of full sequencing of EP4 gene in TO cells revealed a different isoform of EP4 (Fig. 1). We therefore designated the first published variant as As-EP4a while the second identified sequence in TO cells was named as As-EP4b. The length of the As-EP4b was found to be 2346 nucleotides compared to 2423 nucleotides for As-EP4a. While the first half of As-EP4b sequence was found to be identical to As-EP4a, many single nucleotide mutations and segmental deletions were found from nt 1027, which is located almost half through the EP4 gene, and onwards (Fig. 1). The coding region was located from nt 267 to nt 1694 and, although the length of nucleotide for both isoforms were different at C-terminal tail, the encoded length of amino acids were found to be identical, 473 amino acid for both isoforms (Fig. 2). Three amino acids were deleted at positions 389–401 in As-EP4b, whereas another three amino acid deletion was found at position 429–431 As-EP4a. Amino acid sequences for both isoforms were also aligned to EP4 amino acid sequences from several species (Fig. 3). Putative transmembrane domains (TM) 1–7 were noted based on a previous study [10]. Alignment result showed that EP4 genes from Atlantic salmon are in general conserved compared to other species at their seven transmembrane domains while differences in N- and C-terminals were remarkable (Fig. 3). High homology was found between intracellular domain 1 and 2 while in intracellular 3, a deletion of 20–30 amino acids was found only in non-mammalian species (fish and chicken).

The sequence identities between the aligned EP receptors were summarized in Table 2. The transmembrane (TM) domains among all the species are highly conserved. The sequence identity between the two As-EP4 sequences was 91% and they differed mainly in the TM6, third extracellular loop and C-terminal tail. Both sequences share about 71 and 64% to zebrafish EP4a and b respectively, with the highest divergence being in the N-termini, third extracellular loop and C-terminal tail. Notably, high sequence divergence was observed in the third intracellular domain between mammalian and non-mammalian sequences.

**Table 2:** Sequence identities between As-EP4a and other aligned EP4 sequences from different species.

	Identities N-terminal	TM1	Intra1	TM2	Extra1	TM3	Intra2	TM4	Extra2	TM5	Intra3	TM6	Extra3	TM7	C-terminal
As-EP4b	100	100	100	100	100	100	100	100	100	100	100	86	81	95	80
Zf-EP4a	21	76	82	96	62	82	82	63	73	84	75	82	38	86	30
Zf-EP4b	6	90	82	100	69	86	91	79	59	80	82	73	38	95	40
Zf-EP4c	11	55	73	78	69	50	68	26	62	40	30	68	24	76	22
Chicken-EP4	25	81	91	93	54	86	86	79	58	80	69	86	43	90	37
Mouse-EP4	26	70	91	93	46	86	86	79	65	92	46	86	33	95	41
Rat-EP4	26	70	91	93	46	86	86	74	65	92	46	86	33	95	39
Dog-EP4	26	70	91	93	62	86	86	74	65	92	43	86	38	90	40
Cattle-EP4	21	70	91	93	54	86	86	74	65	92	44	86	48	90	41
Human-EP4	32	70	91	93	54	82	86	74	65	88	48	86	38	95	39

### Phylogenetic relationship of EP4 gene among different species

**Fig 4:**
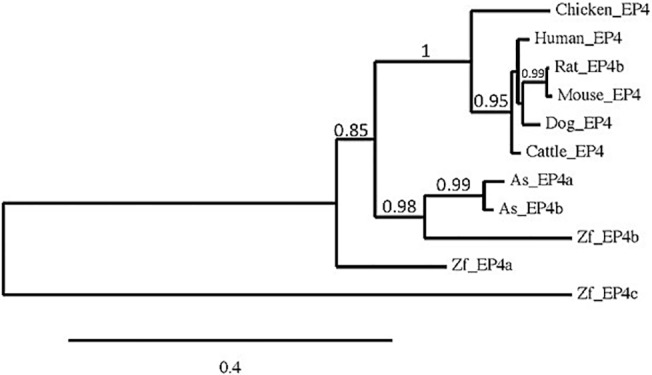
Phylogenetic tree of EP4 receptors from different species. The sequence of Atlantic salmon (As) and zebrafish (Zf) are compared.

To identify the relationship of EP4 receptor sequences from Atlantic salmon and other species, a phylogenetic tree was established using the method as described [27] (Fig. 4). Zebrafish (Zf) EP4c appeared to be the most divergent receptor, distant from all other EP4 receptors. Apart from this, the fish EP4 receptor formed a distinct cluster while the mammalian and chicken EP4s with high bootstrap values formed another cluster. Within the fish cluster, the two As-EP4 isoforms were found to be closer to Zf-EP4b receptor than Zf-EP4a.

### As-EP4a and As-EP4b are differentially distributed in different tissues and TO cells

**Fig 5:**
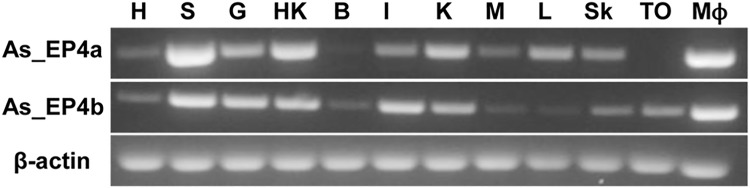
Distribution of EP4a and EP4b in different tissues of salmon. As_EP4a and As_EP4b gene distribution in different tissues are shown. Key: **H**: Heart. **S**: Spleen. **G**: Gill. **HK**: Head Kidney. **B**: Brain, **I**: Intestine, **K**: Kidney, **M**: Muscle, **L**: Liver, **Sk**: Skin, **TO**: TO cell line, **MØ**: Headkidney Macrophages. β-actin gene was used as internal control.

At this point we were not sure whether As-EP4b is an isoform that is naturally present in Atlantic salmon or it is generated during the development of TO cell line. Primers specific for As-EP4a and As-EP4b (Table 1) were therefore designed for PCR, and the expression of the two isoforms in several Atlantic salmon tissues and TO cells was assessed (Fig. 5). All the positive bands seen on the electrophoresis gel were excised and sequenced. The sequence result confirmed that AS-EP4a and AS-EP4b are both present in Atlantic salmon tissues and differentially distributed in the examined tissues (Fig. 5). In general, the mRNA of both isoforms were more abundant in the spleen, gill, intestine, head kidney and kidney while less in liver, heart and skin. EP4a expression was not detectable in the brain while EP4b expression was found to be very low in muscles and liver. Both receptors were expressed in the headkidney macrophages but only AS-EP4b was detected in TO cells (Fig. 5).

### PGE2 modulates LPS induced inflammatory responses in TO cells

**Fig 6:**
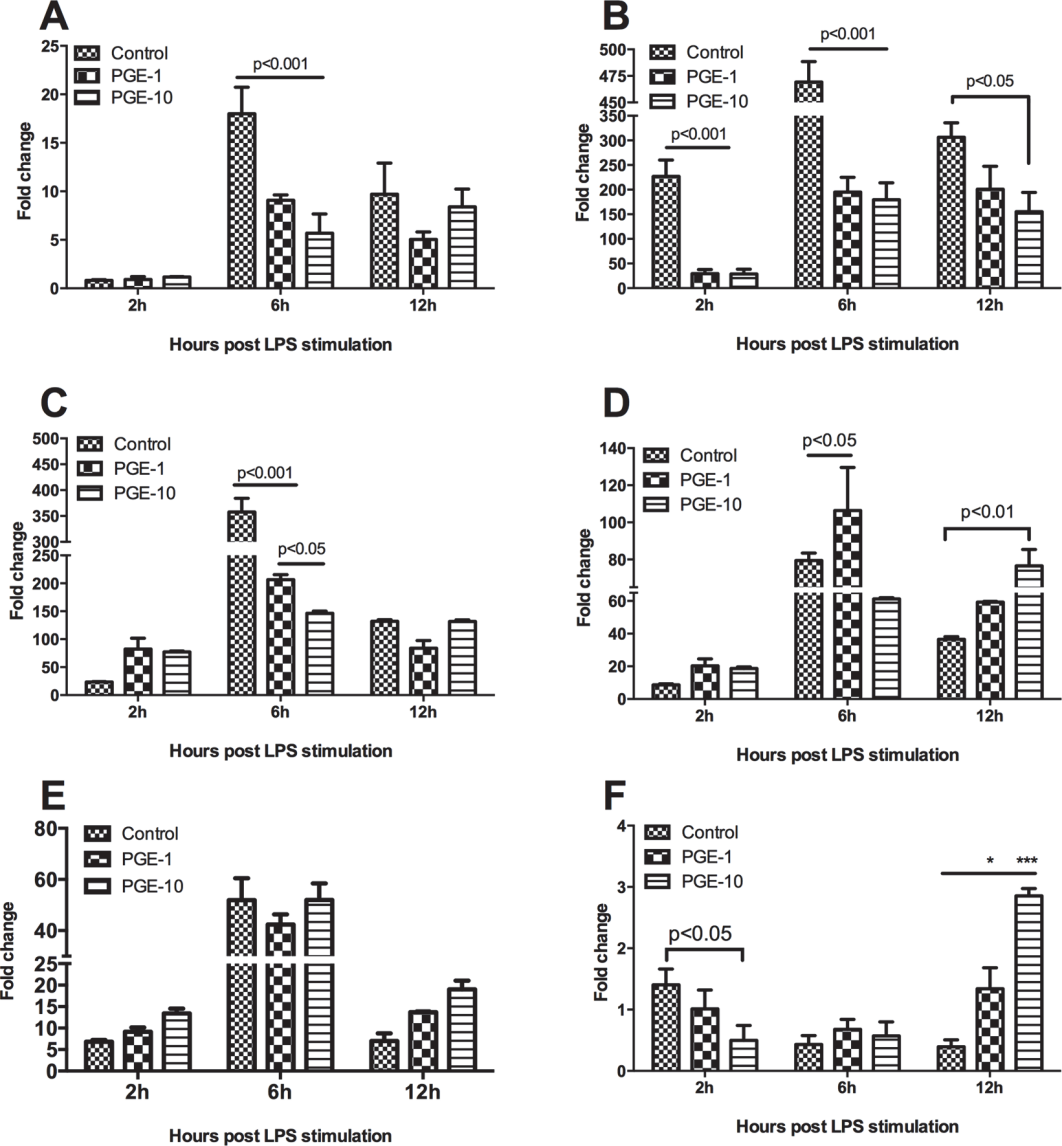
Modulation of responses of defined genes after PGE2 treatment and LPS stimulation in TO cells. Immune response to PGE2 stimulation in TO cells. A) CXCL-10; B) CCL4; C) IL8; D) IL-1β and F) IL6. Different timepoints post LPS stimulation is are indicated. * = p<0.05; ** = p<0.001. Average ± SEM is shown (n = 3).

To study whether PGE2 can modulate immune response in TO cells, TO cells were treated with LPS or pretreated with 1 or 10 μM PGE2) for 1h and subsequently stimulated with LPS. The cells were then incubated for 2, 6, and 12h and sampled for analysis. Stimulation of TO cells with LPS alone resulted in up-regulation of all the examined inflammatory genes (Fig. 6 A-F; control). The up-regulation was most significant at 6 hours post stimulation for all genes, except IL-6. PGE2 pre-incubation lowered the induction of CXCL-10 (Fig. 6A), CCL-4 (Fig. 6B), IL-8 (Fig. 6C) and IL-1β (Fig. 6D) gene expression but had no clear effect on TNF-α (Fig. 6 E). For IL-6 the expression level was lower at 2h but increased beyond control at 12h, seemingly delaying the response at early time while inducing a second wave at later time (Fig. 6F). The induced inhibition was found to be time dependent, with the most pronounced effect being seen at 6 hours post stimulation. Interestingly, CCL-4 (Fig. 6B) was significantly down-regulated by PGE2 at all the examined time points.

### Treatment with EP4 antagonist has no effect of PGE2 induced immunomodulation

**Fig 7:**
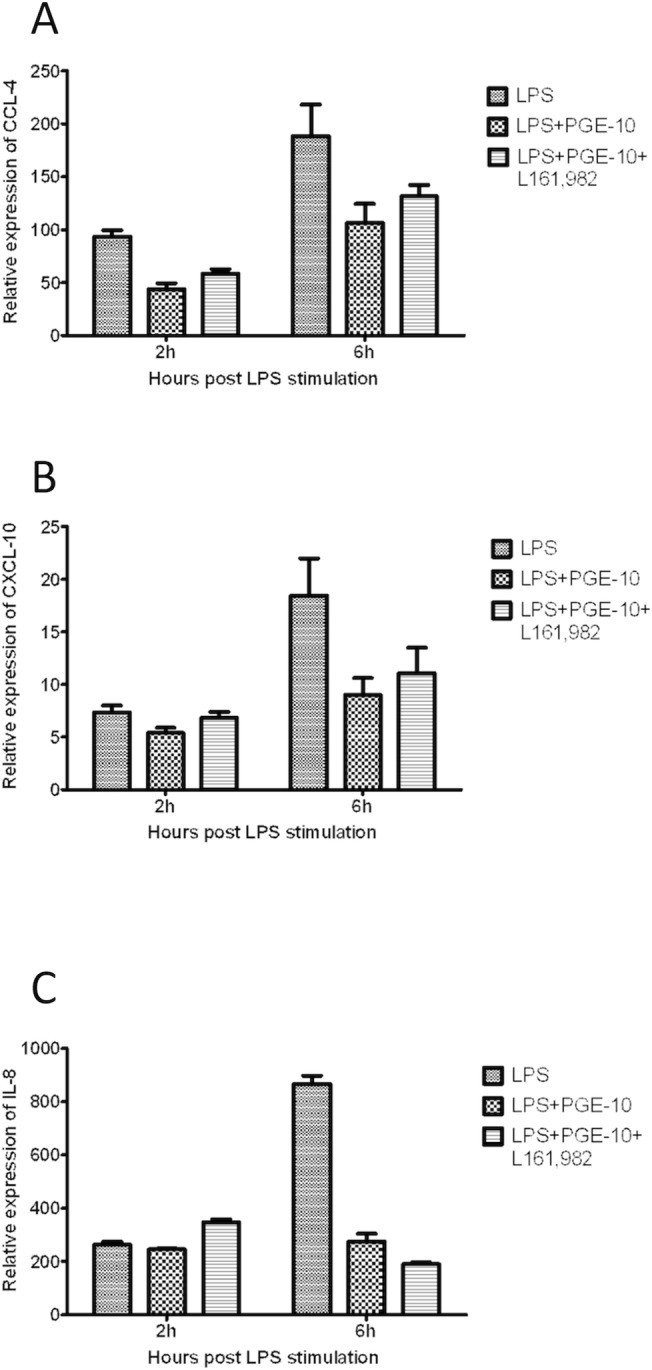
Effect of EP4 antagonist. No significant differences were observed between antagonist L161,982-treated and untreated cells.

To examine whether the immunomodulatory effect induced by PGE2 is mediated through EP4 receptors, TO cells were also pre-treated with EP4 antagonist prior to stimulation with PGE2. No significant differences were observed between antagonist treated and untreated cells (Fig. 7) indicating that EP4 antagonist L161,982 had no effect on the PGE2 induced inhibition of immune responses.

### PGE2 induced immunomodulation in TO cells does not involve cAMP up-regulation

**Fig 8:**
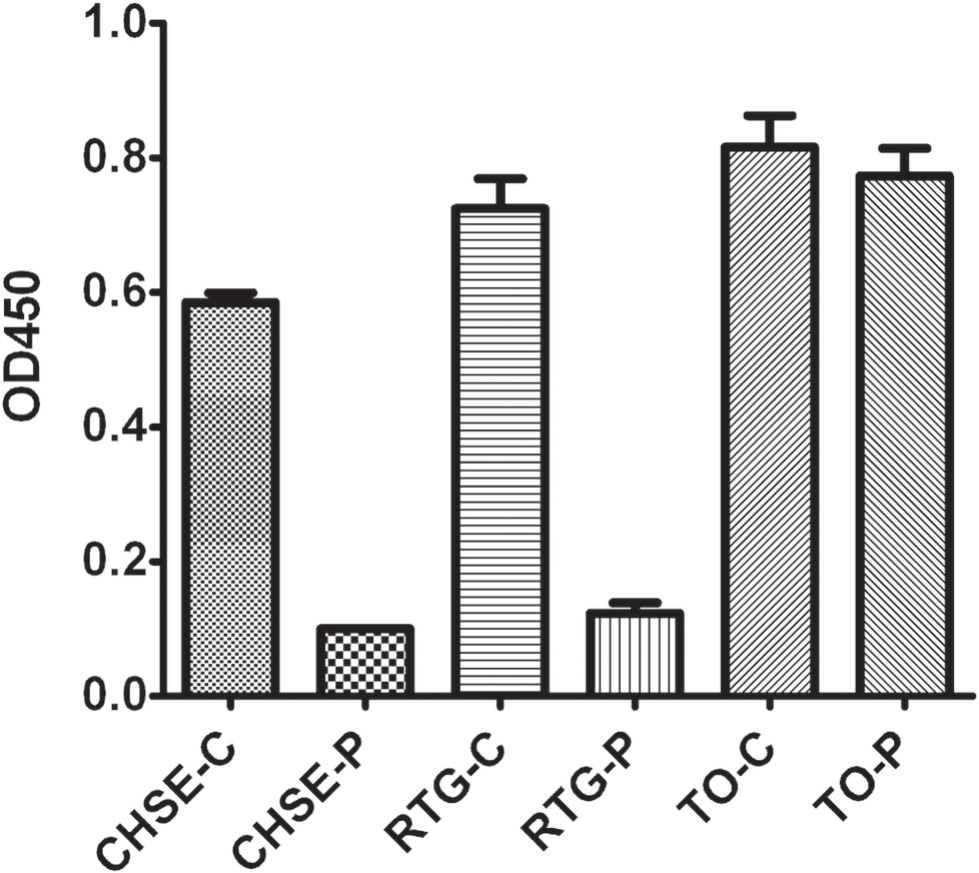
cAMP induction post PGE2 treatment. Three cell lines, CHSE-214, RTG-2 and TO were used in this experiment. Cells were either treated with PGE2 (P) or left without treatment (C).

**Fig 9:**
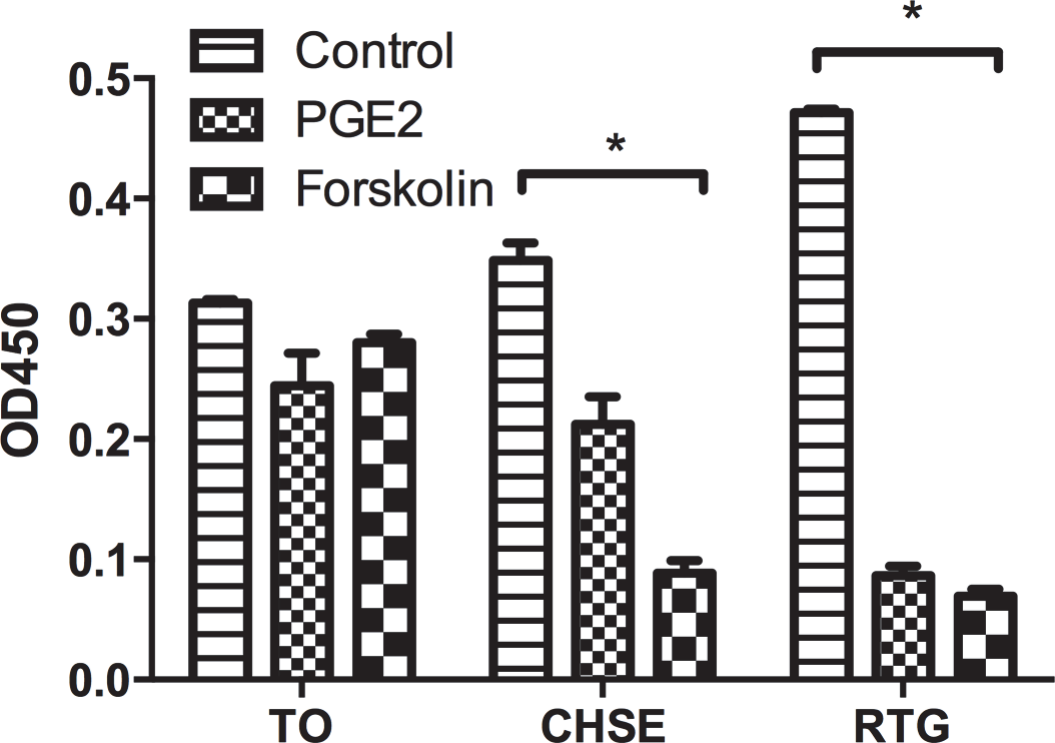
cAMP induction post forskolin treatment. Three cell lines, CHSE-214, RTG-2 and TO were used in this experiment. Cells were either treated with forskolin or left untreated (controls). * = p<0.01 between control and the two treatments. Average ± SEM is shown (n = 3).

To investigate the involvement of cAMP signaling pathway in PGE2 induced effect, the increase of cAMP concentration upon PGE2 treatment was assayed. In TO cells, no significant increase was detected. We included two additional fish-derived cell lines (CHSE-214 and RTG-2 cells, both from salmonid species) and both showed strong cAMP increase in response to PGE2 treatment (Fig. 8). We then included forskolin treatment as a positive control (including all said cells) and found that forskolin results in up-regulation of cAMP in CHSE-214 and RTG-2 while TO cells cAMP levels remained unchanged post stimulation (Fig. 9).

## Discussion

In the present study, we report the identification of a new isoform of the EP4 receptor in Atlantic salmon. The presence of redundant gene copies due to genome duplication has been observed in Atlantic salmon [28]. Some of these copies can be non-functional, but the fact that both identified sequences have the same amino acid length and strong expression in several tissues opt us to propose a new functional isoform of the EP4 receptor. Presence of multiple EP4 isoforms in zebrafish [20] and the difference we found in the C-terminal tail that determines the signaling cascade will be in support for our interpretation. Whether these isoforms are generated by alternative splicing, as for EP3 in higher vertebrates [29,30] or by gene duplication remains to be determined. Further studies are needed, first to identify the functions and the signaling cascade mediated by both isoforms; and secondly to determine whether the functions of these different EP4 isoforms are conserved across fish species. In this regard, comparative studies between fish and mammalian EP4 are required to understand why fish functionally benefit from additional EP4 isoforms. The expression of the mRNA of the different EP4 isoforms in zebrafish was found tissue specific, suggesting that each subtype may mediate specific function [20]. We found that both isoforms are co-expressed in several tissues in Atlantic salmon. This indicates that the functions of the different EP4 isoforms may not be identical across fish species, and that in some species the receptors have evolved to be more specialized than others. Presence of multiple isoforms in one tissue may also serve as a source for functional diversity as different isoforms may have different ligand binding avidity, regulating function by direct competition or have different intrinsic properties such as G protein binding and receptor desensitization [14,30–32].

Duplication in the genome may lead to presence of paralogs of the same genes. Paralogs may have the same function as the ancestral gene, a sub-functionalization in a way where the function of the ancestral gene is divided between the different paralogs or diverge to acquire function that is not present in the ancestral gene [33,34]. Indeed, presence of paralogs has been previously demonstrated in Atlantic salmon [35]. The sequences obtained from different tissues in the current study were very homologous and similar to the previously published EP4 sequence, designated as EP4a in this study [21]. However, based on the presence of some common mutations or deletions they can be divided in three categories: 1) Sequences that are identical to the published sequence, 2) sequences that have 3 nt deletions in the UTR, and 3) sequences that have prolin instead of leucin at residue 61. These sequences may reflect different paralogs especially the two first sequences since they were both found expressed in almost all the examined tissue. Due to the high sequence homology we consider it unlikely that these sequences will have different functions. However, presence of deletion in the regulatory 5’-UTR region, which is important for translation regulation [36], in one of the sequences suggest that a difference in protein expression levels may occur.

The immunomodulatory effect of PGE2 on LPS induced inflammation has been extensively studied in higher vertebrates. In human macrophages, PGE2 was shown to suppress LPS induced mRNA and protein expression of CCL-4, IL-8 [37]. Similarly we found that PGE2 pretreatment lowered LPS induction of both genes and in addition IL-β and CXCL-10 mRNA expression were lowered in TO cells (salmon macrophage cell line). Although the LPS and PGE2 dose used varied between the two studies, the timing when significant inhibition was detected was similar (7 hours for human macrophages and 6 hours for TO cells). These findings are suggestive of PGE2 being an immunomodulator also in salmonid fish, indicating the function is conserved. While PGE2 was also found to impact on IL-6 and TNFα gene expression in mouse peritoneal macrophages during inflammatory response [38], we found no clear effect for PGE2 on the induction of these genes. This difference might be explained by the different inflammatory model used but could also be due to dose effect specially for TNFα since both inhibition and suppression of production can be induced by using different doses of PGE2 [39].

The diverse roles that PGE2 plays in inflammatory response can largely be attributed to the multiple receptors and signaling cascades that can be activated [40,41]. Both EP2 and EP4 were shown to mediate the anti-inflammatory effect induced by PGE2 on LPS-induced inflammation in murine and human macrophages, respectively [37,42]. In the present study we have investigated the role of AS-EP4 receptors in mediating the anti-inflammatory effect of PGE2. While the two characterized EP4 isoforms were present in headkidney macrophages, only EP4b was detected in TO cells. Thus, TO cells can be used as a knock out model to study the differential roles of EP4a and EP4b in mediating PGE2 induced effects. We could not reverse the anti-inflammatory effect induced by PGE2 on LPS induced inflammation by using EP4 antagonist for mammals. There are two possible explanations for this finding; either the effect is not mediated by EP4b or the antagonists does not bind effectively to the AS-EP4b.

The lack of cAMP induction in TO cells after PGE2 stimulation may also be explained by involvement of other variants of PGE2 receptors. The EP4 receptor was initially thought to be coupled only to G_s_α, and therefore induce responses that are mediated only through cAMP signaling, while recent studies have shown that it can also be coupled to G_i_α, which inhibit cAMP production [43]. Further, the involvement of other signaling pathways for EP4 mediated responses has been demonstrated. For example, The EP4 agonist ONO-AE1-329 was shown to attenuate the chemotaxis of human peripheral blood eosinophils independent of cAMP through the phosphatidyl-inositol 3-kinase (PI3K) pathway [44]. The possibility of the anti-inflammatory effect induced by PGE2 in TO cells being mediated through EP4 receptor via cAMP independent pathway, such as PI3K, cannot be ruled out and should be further investigated. Another factor that can explain the lack of cAMP involvement and that would require further investigation is the type of the cAMP generating enzyme adenylyl cyclase (AC) expressed. Some of these ACs are G_s_ sensitive while others are G_i_ sensitive [45] and the absence of G_s_-sensitive isoforms may also lead to lack of cAMP stimulation.
